# Bis(butyl­triethyl­ammonium) di-μ-bromido-bis­[dibromido­mercurate(II)]

**DOI:** 10.1107/S1600536812017011

**Published:** 2012-04-21

**Authors:** Lei Jin

**Affiliations:** a College of Chemistry and Chemical Engineering, Southeast University, Nanjing 210096, People’s Republic of China

## Abstract

In the title mol­ecular salt, (C_10_H_24_N)_2_[Hg_2_Br_6_], the complete anion is generated by crystallographic inversion symmetry, forming a pair of edge-sharing HgBr_4_ tetra­hedra. In the crystal, the cations and anions are linked by weak C—H⋯Br inter­actions.

## Related literature

For a related structure and background to mol­ecular ferroelectrics, see: Jin (2012 ▶).
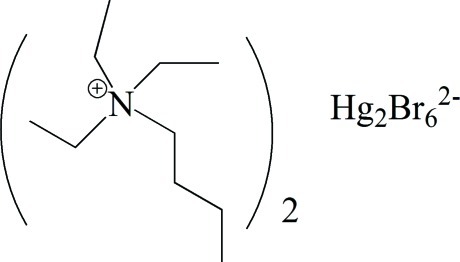


## Experimental

### 

#### Crystal data

(C_10_H_24_N)_2_[Hg_2_Br_6_]
*M* *_r_* = 1197.24
Triclinic, 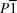

*a* = 7.6372 (15) Å
*b* = 10.318 (2) Å
*c* = 11.185 (2) Å
α = 76.70 (3)°
β = 72.22 (3)°
γ = 85.57 (3)°
*V* = 816.8 (3) Å^3^
*Z* = 1
Mo *K*α radiation
μ = 16.74 mm^−1^
*T* = 293 K
0.28 × 0.24 × 0.20 mm

#### Data collection

Rigaku Mercury2 diffractometer
Absorption correction: multi-scan (*CrystalClear*; Rigaku, 2005 ▶) *T*_min_ = 0.013, *T*_max_ = 0.035
7659 measured reflections
3209 independent reflections
2596 reflections with *I* > 2σ(*I*)
*R*_int_ = 0.053

#### Refinement

*R*[*F* ^2^ > 2σ(*F* ^2^)] = 0.056
*wR*(*F* ^2^) = 0.151
*S* = 1.05
3209 reflections
141 parameters
H-atom parameters constrained
Δρ_max_ = 1.27 e Å^−3^
Δρ_min_ = −1.83 e Å^−3^

### 

Data collection: *CrystalClear* (Rigaku, 2005 ▶); cell refinement: *CrystalClear*; data reduction: *CrystalClear*; program(s) used to solve structure: *SHELXS97* (Sheldrick, 2008 ▶); program(s) used to refine structure: *SHELXL97* (Sheldrick, 2008 ▶); molecular graphics: *SHELXTL* (Sheldrick, 2008 ▶); software used to prepare material for publication: *SHELXTL*.

## Supplementary Material

Crystal structure: contains datablock(s) I, global. DOI: 10.1107/S1600536812017011/hb6722sup1.cif

Structure factors: contains datablock(s) I. DOI: 10.1107/S1600536812017011/hb6722Isup2.hkl

Additional supplementary materials:  crystallographic information; 3D view; checkCIF report

## Figures and Tables

**Table:** 

Hg1—Br2	2.4963 (18)
Hg1—Br3	2.5059 (17)
Hg1—Br1^i^	2.681 (2)
Hg1—Br1	2.7092 (19)

**Table:** 

Hg1^i^—Br1—Hg1	88.53 (5)

Symmetry code: (i) 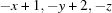.

**Table 2:** Hydrogen-bond geometry (Å, °)

*D*—H⋯*A*	*D*—H	H⋯*A*	*D*⋯*A*	*D*—H⋯*A*
C3—H3*B*⋯Br1^ii^	0.97	2.91	3.837 (15)	160
C6—H6*A*⋯Br2	0.96	3.00	3.833 (16)	147
C7—H7*B*⋯Br2	0.97	3.03	3.973 (13)	165

Symmetry code: (ii) 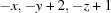.

**Fig. 1.**
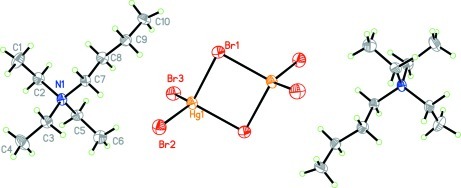
The mlecular structure of the title compound, showing 30% probability displacement ellipsoids. Unlabelled atoms are related to the labelled atoms by the (-x, -y + 2, -z + 1) symmetry transformation.

## supplementary crystallographic information

### Comment

Asa part of our studies (Jin, 2012) of molecular salts with possible ferroelectric properties, the title compound has been synthesized and its crystal structure is herein reported.

The title compound, (C_10_H_16_N^+^)_2_^.^Hg_2_Br_6_^2-^ crystallizes in the triclinic P-1 space group, and the structure of title compound contains isolated bitetrahedral [Hg_2_Br_6_]^2-^ units,which consisiting of two distorted tetrahedral sharing one common edge and two butyltriethylammonium cations (Fig 1). The terminal bond distance of Hg–Br being 2.4963 (18)Å and 2.5059 (17)Å, the bond angles of Br–Hg–Br being in the range from 107.16 (6)° to 122.48 (7)°; the bridging are in the range 2.681 (2)Å and 2.7092 (19)Å, and the bond angles of Br–Hg–Br varying from 107.16 (6)° to 113.07 (7)°, thus deviating from ideal tetrahedral angles of 109.5°. An inversion centre is located an the centre of the [Hg_2_Br_6_]^2-^ unit,and the bridge distance of Br–Br is 3.860Å.

In the crystal, there are weak C—H···Br hydrogen bonds (Table 1), which link the cations and anions into a three-dimensional network.

### Experimental

In room temperature butyltriethylammonium (5 mmol,1.17 g) in 20 ml water, then a water solution with HgBr_2_ (5 mmol, 1.36 g) was dropped slowly into the previous solution with properly sirring. Colourless blocks were obtained by the slow evaporation of the above solution after one week in air with some colorless solid blocks appeared after days.

The dielectric constant of the compound as a function of temperature indicates that the permittivity is basically temperature-independent (ε = C/(T–T_0_)), indicating that this compound is not ferroelectric over the measured temperature range (below the melting point).

### Refinement

H atoms were placed in calculated positions(C—H = 0.96Å and 0.97 Å for C*sp^3^* atoms), assigned fixed *U*_iso_ values [*U*_iso_ = 1.2*U*eq(C*sp^2^*/N) and 1.5*U*eq(C*sp^3^*)] and allowed to ride.

### Crystal data

**Table:**

(C_10_H_24_N)_2_[Hg_2_Br_6_]	*V* = 816.8 (3) Å^3^
*M**_r_* = 1197.24	*Z* = 1
Triclinic, *P*1	*F*(000) = 552
Hall symbol: -P 1	*D*_x_ = 2.434 Mg m^−^^3^
*a* = 7.6372 (15) Å	Mo *K*α radiation, λ = 0.71073 Å
*b* = 10.318 (2) Å	θ = 3.1–26°
*c* = 11.185 (2) Å	µ = 16.74 mm^−^^1^
α = 76.70 (3)°	*T* = 293 K
β = 72.22 (3)°	Block, colorless
γ = 85.57 (3)°	0.28 × 0.24 × 0.20 mm

### Data collection

**Table:**

Rigaku Mercury2 diffractometer	3209 independent reflections
Radiation source: fine-focus sealed tube	2596 reflections with *I* > 2σ(*I*)
Graphite monochromator	*R*_int_ = 0.053
Detector resolution: 13.6612 pixels mm^-1^	θ_max_ = 26.0°, θ_min_ = 3.1°
CCD_Profile_fitting scans	*h* = −9→9
Absorption correction: multi-scan (*CrystalClear*; Rigaku, 2005)	*k* = −12→12
*T*_min_ = 0.013, *T*_max_ = 0.035	*l* = −13→13
7659 measured reflections	

### Refinement

**Table:**

Refinement on *F*^2^	Secondary atom site location: difference Fourier map
Least-squares matrix: full	Hydrogen site location: inferred from neighbouring sites
*R*[*F*^2^ > 2σ(*F*^2^)] = 0.056	H-atom parameters constrained
*wR*(*F*^2^) = 0.151	*w* = 1/[σ^2^(*F*_o_^2^) + (0.0572*P*)^2^ + 12.6923*P*] where *P* = (*F*_o_^2^ + 2*F*_c_^2^)/3
*S* = 1.05	(Δ/σ)_max_ < 0.001
3209 reflections	Δρ_max_ = 1.27 e Å^−^^3^
141 parameters	Δρ_min_ = −1.83 e Å^−^^3^
0 restraints	Extinction correction: *SHELXL97* (Sheldrick, 2008), Fc^*^=kFc[1+0.001xFc^2^λ^3^/sin(2θ)]^-1/4^
Primary atom site location: structure-invariant direct methods	Extinction coefficient: 0.0069 (8)

### Special details

**Table:**

Geometry. All e.s.d.'s (except the e.s.d. in the dihedral angle between two l.s. planes) are estimated using the full covariance matrix. The cell e.s.d.'s are taken into account individually in the estimation of e.s.d.'s in distances, angles and torsion angles; correlations between e.s.d.'s in cell parameters are only used when they are defined by crystal symmetry. An approximate (isotropic) treatment of cell e.s.d.'s is used for estimating e.s.d.'s involving l.s. planes.
Refinement. Refinement of *F*^2^ against ALL reflections. The weighted *R*-factor *wR* and goodness of fit *S* are based on *F*^2^, conventional *R*-factors *R* are based on *F*, with *F* set to zero for negative *F*^2^. The threshold expression of *F*^2^ > σ(*F*^2^) is used only for calculating *R*-factors(gt) *etc*. and is not relevant to the choice of reflections for refinement. *R*-factors based on *F*^2^ are statistically about twice as large as those based on *F*, and *R*- factors based on ALL data will be even larger.

### Fractional atomic coordinates and isotropic or equivalent isotropic displacement parameters (Å^2^)

**Table:**

	*x*	*y*	*z*	*U*_iso_*/*U*_eq_	
Hg1	0.46437 (8)	0.89173 (5)	0.16554 (5)	0.0447 (3)	
Br1	0.2377 (2)	1.01250 (17)	0.02768 (16)	0.0588 (4)	
Br2	0.4361 (3)	1.01131 (17)	0.34093 (17)	0.0650 (5)	
Br3	0.4370 (3)	0.64383 (15)	0.20690 (19)	0.0661 (5)	
N1	0.0814 (15)	0.6872 (9)	0.7024 (10)	0.034 (2)	
C7	0.0311 (17)	0.7828 (12)	0.5918 (12)	0.036 (3)	
H7A	−0.0722	0.8369	0.6282	0.043*	
H7B	0.1340	0.8421	0.5455	0.043*	
C5	0.2418 (18)	0.5990 (12)	0.6507 (14)	0.042 (3)	
H5A	0.2652	0.5351	0.7228	0.051*	
H5B	0.2068	0.5495	0.5978	0.051*	
C8	−0.018 (2)	0.7233 (13)	0.4966 (14)	0.047 (3)	
H8A	−0.1218	0.6645	0.5409	0.056*	
H8B	0.0852	0.6705	0.4573	0.056*	
C9	−0.066 (2)	0.8301 (14)	0.3919 (14)	0.047 (3)	
H9A	−0.1715	0.8804	0.4318	0.056*	
H9B	0.0364	0.8911	0.3512	0.056*	
C3	0.128 (2)	0.7737 (13)	0.7835 (13)	0.045 (3)	
H3A	0.2291	0.8319	0.7292	0.054*	
H3B	0.0227	0.8295	0.8124	0.054*	
C2	−0.080 (2)	0.5964 (13)	0.7817 (13)	0.047 (3)	
H2A	−0.1085	0.5471	0.7259	0.056*	
H2B	−0.0419	0.5323	0.8474	0.056*	
C10	−0.109 (2)	0.7763 (16)	0.2901 (15)	0.056 (4)	
H10A	−0.0130	0.7158	0.2587	0.084*	
H10B	−0.1167	0.8485	0.2204	0.084*	
H10C	−0.2239	0.7302	0.3261	0.084*	
C1	−0.249 (2)	0.6613 (15)	0.8449 (17)	0.062 (4)	
H1A	−0.2266	0.7043	0.9062	0.094*	
H1B	−0.3435	0.5958	0.8882	0.094*	
H1C	−0.2884	0.7264	0.7816	0.094*	
C6	0.417 (2)	0.6716 (16)	0.5727 (16)	0.059 (4)	
H6A	0.3979	0.7321	0.4985	0.088*	
H6B	0.5118	0.6085	0.5455	0.088*	
H6C	0.4543	0.7206	0.6241	0.088*	
C4	0.181 (3)	0.6951 (17)	0.9002 (17)	0.073 (5)	
H4A	0.0940	0.6253	0.9458	0.109*	
H4B	0.1817	0.7534	0.9558	0.109*	
H4C	0.3017	0.6570	0.8726	0.109*	

### Atomic displacement parameters (Å^2^)

**Table:**

	*U*^11^	*U*^22^	*U*^33^	*U*^12^	*U*^13^	*U*^23^
Hg1	0.0577 (4)	0.0362 (3)	0.0401 (3)	−0.0008 (2)	−0.0150 (2)	−0.0074 (2)
Br1	0.0477 (8)	0.0687 (10)	0.0574 (10)	0.0021 (7)	−0.0160 (7)	−0.0090 (8)
Br2	0.0730 (11)	0.0624 (10)	0.0602 (10)	−0.0025 (8)	−0.0153 (8)	−0.0200 (8)
Br3	0.0729 (11)	0.0390 (8)	0.0865 (12)	−0.0005 (7)	−0.0275 (9)	−0.0085 (8)
N1	0.050 (6)	0.019 (4)	0.034 (5)	−0.002 (4)	−0.012 (5)	−0.004 (4)
C7	0.040 (7)	0.034 (6)	0.035 (7)	−0.005 (5)	−0.011 (6)	−0.008 (5)
C5	0.040 (7)	0.031 (6)	0.054 (8)	0.009 (5)	−0.020 (6)	−0.002 (6)
C8	0.056 (8)	0.030 (6)	0.054 (9)	−0.004 (6)	−0.021 (7)	−0.003 (6)
C9	0.044 (7)	0.044 (7)	0.046 (8)	0.004 (6)	−0.017 (6)	0.005 (6)
C3	0.059 (8)	0.036 (7)	0.042 (8)	−0.015 (6)	−0.016 (7)	−0.009 (6)
C2	0.059 (9)	0.036 (7)	0.040 (8)	−0.014 (6)	−0.013 (7)	0.008 (6)
C10	0.060 (9)	0.057 (9)	0.059 (10)	−0.003 (7)	−0.035 (8)	−0.004 (7)
C1	0.056 (9)	0.047 (8)	0.068 (11)	−0.014 (7)	0.009 (8)	−0.013 (8)
C6	0.054 (9)	0.054 (9)	0.063 (10)	0.014 (7)	−0.016 (8)	−0.010 (8)
C4	0.110 (15)	0.059 (10)	0.058 (10)	−0.015 (10)	−0.048 (11)	0.005 (8)

### Geometric parameters (Å, º)

**Table:**

Hg1—Br2	2.4963 (18)	C9—H9B	0.9700
Hg1—Br3	2.5059 (17)	C3—C4	1.52 (2)
Hg1—Br1^i^	2.681 (2)	C3—H3A	0.9700
Hg1—Br1	2.7092 (19)	C3—H3B	0.9700
Br1—Hg1^i^	2.681 (2)	C2—C1	1.46 (2)
N1—C5	1.517 (16)	C2—H2A	0.9700
N1—C7	1.524 (15)	C2—H2B	0.9700
N1—C2	1.522 (16)	C10—H10A	0.9600
N1—C3	1.538 (15)	C10—H10B	0.9600
C7—C8	1.492 (18)	C10—H10C	0.9600
C7—H7A	0.9700	C1—H1A	0.9600
C7—H7B	0.9700	C1—H1B	0.9600
C5—C6	1.50 (2)	C1—H1C	0.9600
C5—H5A	0.9700	C6—H6A	0.9600
C5—H5B	0.9700	C6—H6B	0.9600
C8—C9	1.526 (18)	C6—H6C	0.9600
C8—H8A	0.9700	C4—H4A	0.9600
C8—H8B	0.9700	C4—H4B	0.9600
C9—C10	1.50 (2)	C4—H4C	0.9600
C9—H9A	0.9700		
			
Br2—Hg1—Br3	122.48 (7)	C4—C3—N1	114.4 (11)
Br2—Hg1—Br1^i^	107.16 (6)	C4—C3—H3A	108.7
Br3—Hg1—Br1^i^	113.07 (7)	N1—C3—H3A	108.7
Br2—Hg1—Br1	108.17 (6)	C4—C3—H3B	108.7
Br3—Hg1—Br1	110.02 (6)	N1—C3—H3B	108.7
Br1^i^—Hg1—Br1	91.47 (5)	H3A—C3—H3B	107.6
Hg1^i^—Br1—Hg1	88.53 (5)	C1—C2—N1	116.4 (11)
C5—N1—C7	110.3 (9)	C1—C2—H2A	108.2
C5—N1—C2	107.3 (9)	N1—C2—H2A	108.2
C7—N1—C2	109.7 (10)	C1—C2—H2B	108.2
C5—N1—C3	111.9 (10)	N1—C2—H2B	108.2
C7—N1—C3	106.6 (8)	H2A—C2—H2B	107.3
C2—N1—C3	111.1 (10)	C9—C10—H10A	109.5
C8—C7—N1	117.3 (10)	C9—C10—H10B	109.5
C8—C7—H7A	108.0	H10A—C10—H10B	109.5
N1—C7—H7A	108.0	C9—C10—H10C	109.5
C8—C7—H7B	108.0	H10A—C10—H10C	109.5
N1—C7—H7B	108.0	H10B—C10—H10C	109.5
H7A—C7—H7B	107.2	C2—C1—H1A	109.5
C6—C5—N1	114.9 (11)	C2—C1—H1B	109.5
C6—C5—H5A	108.5	H1A—C1—H1B	109.5
N1—C5—H5A	108.5	C2—C1—H1C	109.5
C6—C5—H5B	108.5	H1A—C1—H1C	109.5
N1—C5—H5B	108.5	H1B—C1—H1C	109.5
H5A—C5—H5B	107.5	C5—C6—H6A	109.5
C7—C8—C9	111.7 (11)	C5—C6—H6B	109.5
C7—C8—H8A	109.3	H6A—C6—H6B	109.5
C9—C8—H8A	109.3	C5—C6—H6C	109.5
C7—C8—H8B	109.3	H6A—C6—H6C	109.5
C9—C8—H8B	109.3	H6B—C6—H6C	109.5
H8A—C8—H8B	107.9	C3—C4—H4A	109.5
C10—C9—C8	114.1 (12)	C3—C4—H4B	109.5
C10—C9—H9A	108.7	H4A—C4—H4B	109.5
C8—C9—H9A	108.7	C3—C4—H4C	109.5
C10—C9—H9B	108.7	H4A—C4—H4C	109.5
C8—C9—H9B	108.7	H4B—C4—H4C	109.5
H9A—C9—H9B	107.6		

Symmetry code: (i) −*x*+1, −*y*+2, −*z*.

### Hydrogen-bond geometry (Å, º)

**Table:**

*D*—H···*A*	*D*—H	H···*A*	*D*···*A*	*D*—H···*A*
C3—H3*B*···Br1^ii^	0.97	2.91	3.837 (15)	160
C6—H6*A*···Br2	0.96	3.00	3.833 (16)	147
C7—H7*B*···Br2	0.97	3.03	3.973 (13)	165

Symmetry code: (ii) −*x*, −*y*+2, −*z*+1.
